# Comparative Metabolic Profiling of Grape Pulp during the Growth Process Reveals Systematic Influences under Root Restriction

**DOI:** 10.3390/metabo11060377

**Published:** 2021-06-11

**Authors:** Feng Leng, Shuyan Duan, Shiren Song, Liping Zhao, Wenping Xu, Caixi Zhang, Chao Ma, Lei Wang, Shiping Wang

**Affiliations:** 1 Department of Plant Science, School of Agriculture and Biology, Shanghai Jiao Tong University, Shanghai 200240, China; lengfeng@yzu.edu.cn (F.L.); hongloudsy@sjtu.edu.cn (S.D.); sr.song@sjtu.edu.cn (S.S.); lpzhao07@sjtu.edu.cn (L.Z.); wp-xu@sjtu.edu.cn (W.X.); acaizh@sjtu.edu.cn (C.Z.); chaoma2015@sjtu.edu.cn (C.M.); fruit@sjtu.edu.cn (S.W.); 2 College of Horticulture and Plant Protection, Yangzhou University, Yangzhou 225009, China; 3 Key Laboratory of Agro-Products Processing Technology of Shandong, Institute of Agro-Food Science and Technology, Shandong Academy of Agricultural Sciences, Jinan 250100, China

**Keywords:** *Vitis* *vinifera* L., root restriction, metabolomics, pulp tissue, fruit quality

## Abstract

The compositions and contents of metabolites in the pulp tissue play critical roles in the fruit quality for table grape. In this study, the effects of root restriction (RR) on the primary and secondary metabolites of pulp tissue at five developmental stages were studied at the metabolomics level, using “Red Alexandria” grape berry (*Vitis vinifera* L.) as materials. The main results were as follows: 283 metabolites were annotated by using ultra-high performance liquid chromatography-mass spectrometry (UPLC-MS); 28 and 16 primary metabolites contents were increased and decreased, and 11 and 19 secondary metabolites contents were increased and decreased, respectively, along the berry development; RR significantly decreased 12 metabolites (four amino acids and derivatives, three organic acids, four flavonoids and one other compound) contents, and improved 40 metabolites (22 amino acids and derivatives, six nucleotides, four carbohydrates, four cofactors, three cinnamic acids and one other compound) accumulation at the different developmental stages. Altogether, our study would be helpful to increase our understanding of grape berry’s responses to RR stress.

## 1. Introduction

Grapevine (*Vitis vinifera* L.) as a non-climacteric and important economical fruit is cultivated worldwide [1,2]. The grape berry follows a double sigmoidal growth curve with three major stages, and each stage undergoes complex series changes of metabolites and gene expression [3]. The metabolites in the grape berries are mainly divided into two types, primary and secondary metabolites, and many of these metabolites show beneficial effects on human health [4]. Sugars, organic acids and amino acids are the main primary metabolites, which are mainly accumulated in pulp tissues. Moreover, most of the secondary metabolites, including phenolic acids and flavonoids, are also mainly found in the skin tissues [5,6]. A range of biotic and abiotic stresses, such as water deficit [7,8,9], temperature stress [10,11], solar irradiance [6,12], phytohormones [12,13] and pathogen infection [14] easily affects the compositions and contents of these metabolites during the grape berry development and ripening stages.

Root restriction (RR) as a type of stress is a novel cultivation technique by restricting the root growth in a certain volume to improve the utilizing efficiency of agricultural resources [2,15]. Previous reports demonstrated that RR treatment limits the shoots and roots growth, increases the sugar content and the total and individual anthocyanin levels, promotes ascorbic acid and carotenoids accumulation, and accelerates the grape ripening process by changes in different phytohormones levels compared with the traditional cultivation (RC) [15,16,17,18,19,20]. Recently, our team annotated 291 metabolites in the table grape ‘Red Alexandria’ skin tissue while studying the growth processes using a non-targeted metabolomics method. We found that RR significantly influences secondary metabolism, particularly at the pre-veraison stage, and advances coloring of the berries [21].

“Red Alexandria” is a kind of table grape whose pulp tissue plays a vital role in its fruit quality. Therefore, in this study, we applied non-targeted metabolomics to comparatively investigate the effects of RR on the pulp tissue metabolites in “Red Alexandria” grape during growth processes, to find evidence that RR improves fruit quality and provides theoretical basis for production practice.

## 2. Results

### 2.1. Metabolite Profiling of Grape Pulp Samples

In the present study, a total of 283 metabolites were annotated in the grape pulp from more than 1000 characteristic features by using UPLC-MS. Among them were 157 primary metabolites, including 48 carbohydrates and organic acids, 51 amino acids and derivatives, 19 nucleotides, 28 lipids and 11 CPGECs (cofactors, prosthetic groups, and electron carriers), 107 secondary metabolites, and 19 other compounds (Supplementary Table S1). Notably, isoscoparin, malvidin-3-*O*-(6-*O*-coumaryl)-glucoside and uridine were detected after veraison, while naringenin was only found at the ripening stage. Besides that, pheophorbide A and tricin 4’-*O*-(syringyl alcohol) ether *O*-hexoside were not found in the RC samples, cysteine, maltotriose and isorhamnetin-*O*-rhamnoside were not found in S1 samples of RC, while 4-acetamidobutanoate and isorhamnetin-*O*-rhamnoside were not found in S5 samples of RR.

### 2.2. Kinetic Patterns of Developing Grape Pulp Metabolomes

**Figure 1:**
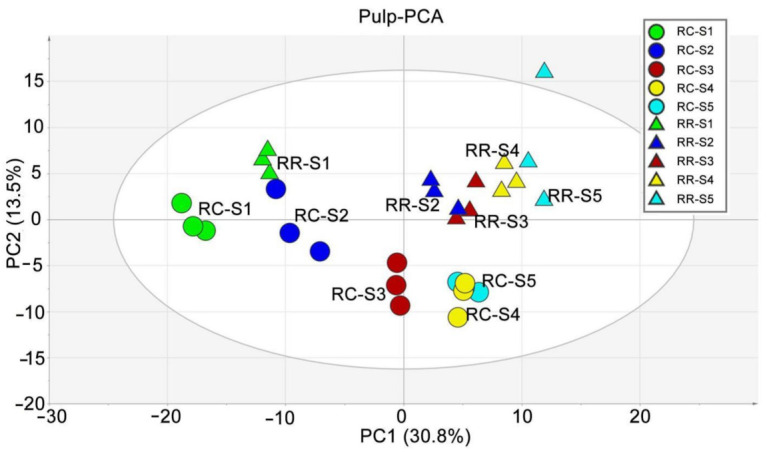
PCA of pulp at different berry developmental stages. Traditional cultivation (RC) and root restriction (RR) cultivation are represented by circles and triangles, respectively. Green, blue, brown, yellow, and cyan represent samples collected at S1, S2, S3, S4, S5 stage, respectively.

The unsupervised multivariate data analysis of the annotated 283 metabolites was performed using principal component analysis (PCA) to observe the kinetic patterns of developing grape pulp metabolomes (Figure 1). Our study found that the separation between different cultivation methods in the same stage and between different developmental stages of the same cultivation method was clear, indicating that the metabolic variations of pulp had a kinetic pattern dependent on the developmental period and cultivation methods. The first two principal components (PCs) explained 44.3% of the total variance of pulp metabolism (30.8% for PC1 and 13.5% for PC2). PC1 and PC2 separated the variations by developmental stages and cultivation methods, respectively. The results of principal component analysis showed that the effect of the development period on pulp metabolites was greater than that of the cultivation methods. Interestingly, from PCA plots, the distance between different stages becomes smaller with fruit development, indicating that the difference between metabolites in pulp becomes smaller with fruit development.

### 2.3. Metabolic Changes of Grape Pulp at Different Developmental Stages

**Figure 2:**
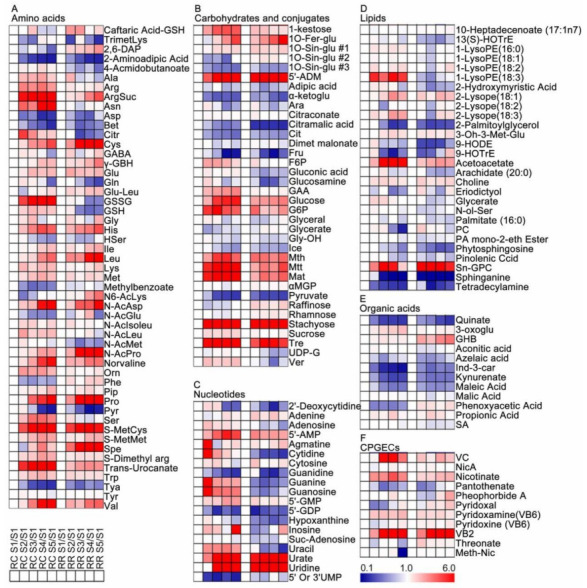
Heatmap of the primary metabolite changes in pulp tissues of developing berries for each cultivation method as compared with S1 stage. (**A**) Amino acids and derivatives; (**B**) carbohydrates and conjugates; (**C**) nucleotides; (**D**) lipids; (**E**) organic acids; (**F**) CPGECs. RR and RC are the abbreviations of root restriction and traditional cultivation, respectively. A *t*-test was performed for statistical analysis. Each data was the mean value of three biological replicates. The full names of metabolites are attached to Supplementary Table S1.

To discover the metabolite variations of grape pulp at different developmental stages, we compared 283 metabolites at a given stage of RR or RC with the corresponding S1 stage, respectively. To discriminate, *t*-test and false discovery rate (FDR) analysis were used. Compared with the S1 stage, the contents of 28 primary metabolites were increased in each developmental stage, including 14 amino acids and derivatives, five carbohydrates, two lipids, three nucleotides, two cofactors and two organic acids, such as cysteine, gamma-guanidinobutyric acid, histidine, proline, riboflavin, urate and uridine, etc. While the contents of 16 primary metabolites were decreased at each developmental stage, including two amino acids and derivatives, four carbohydrates, two lipids, four nucleotides and four organic acids, such as 2-aminoadipic acid, alpha-ketoglutarate, citrate, pyruvate, cytidine and quinate, etc. Other metabolites were increased or decreased at different stages during the berry development (Figure 2, Supplementary Table S2).

**Figure 3:**
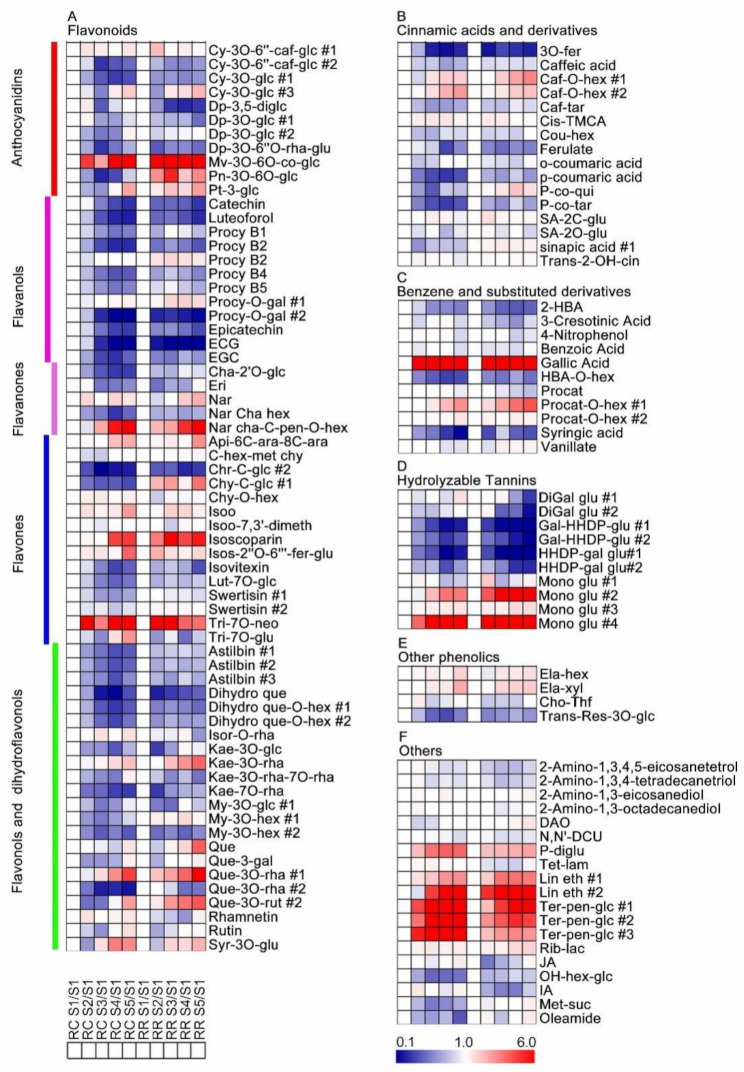
Heatmap of the secondary metabolite changes in pulp tissues of developing berries for each cultivation method as compared with S1 stage. (**A**) Flavonoids, including anthocyanins, flavanols, flavanones, flavones and flavonols and dihydraflavonols; (**B**) cinnamic acids and derivatives; (**C**) benzene and substituted derivatives; (**D**) hydrolysable tannins; (**E**) other phenolics; (**F**) others. RR and RC are the abbreviations of root restriction and traditional cultivation, respectively. A *t*-test was performed for statistical analysis. Each data was the mean value of three biological replicates. The full names of metabolites are attached to Supplementary Table S1.

Compared with the S1 stage, 11 and 19 secondary metabolites consistently increased and decreased, respectively, along with berry development. Some metabolites were elevated before veraison and showed no significant difference along berry development, such as salicylic acid 2-C-β-D-glucopyranoside, chrysoeriol C-glucoside #2 and peonidin-3-*O*-(6-*O*-coumaryl)-glucoside, etc. Some metabolites did not begin to rise until after the S3 stage, such as caffeic acid-*O*-hexoside #1 and linoleoyl ethanolamide #2 (Figure 3, Supplementary Table S2).

**Figure 4:**
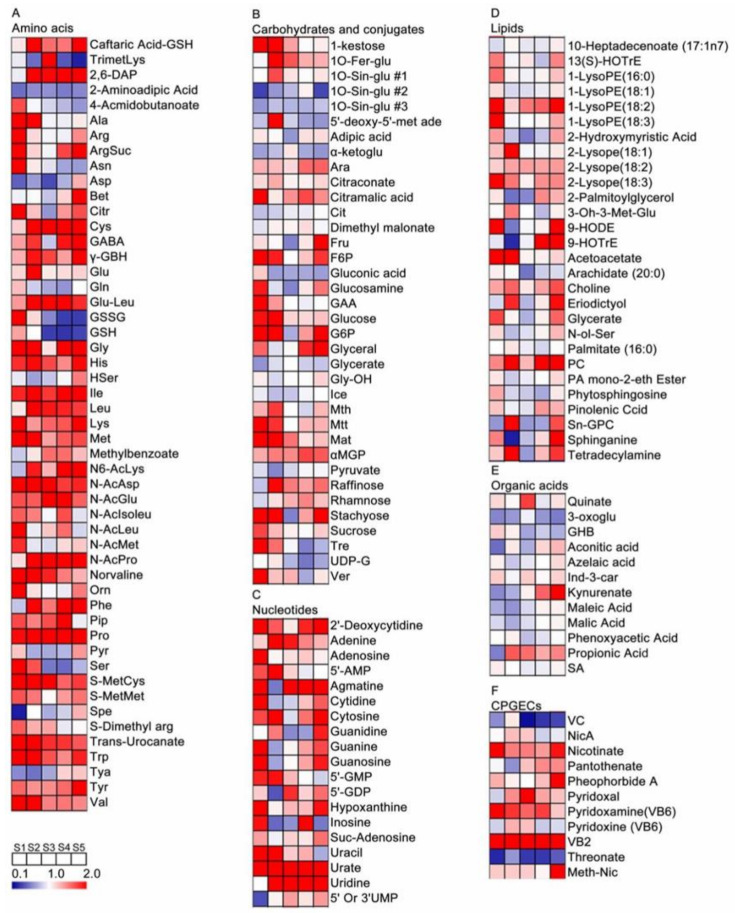
Heatmap of the primary metabolite changes in pulp of developing berries under RR cultivation as compared with those in RC (RR vs. RC). (**A**) Amino acids and derivatives; (**B**) carbohydrates and conjugates; (**C**) nucleotides; (**D**) lipids; (**E**) organic acids; (**F**) CPGECs. RR and RC are the abbreviations of root restriction and traditional cultivation, respectively. A *t*-test was performed for statistical analysis. Each data was the mean value of three biological replicates. The full names of metabolites are attached to Supplementary Table S1.

**Figure 5:**
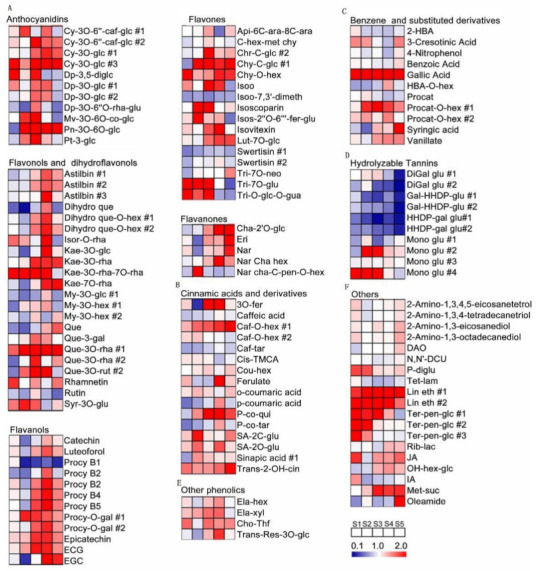
Heatmap of the secondary metabolite changes in pulp of developing berries under RR cultivation as compared with those in RC (RR vs. RC). (**A**) Flavonoids, including anthocyanins, flavanols, flavanones, flavones and flavonols and dihydraflavonols; (**B**) cinnamic acids and derivatives; (**C**) benzene and substituted derivatives; (**D**) hydrolysable tannins; (**E**) other phenolics; (**F**) others. RR and RC are the abbreviations of root restriction and traditional cultivation, respectively. A *t*-test was performed for statistical analysis. Each data was the mean value of three biological replicates. The full names of metabolites are attached to Supplementary Table S1.

In order to study the effects of RR on grape pulp metabolites, a heatmap of metabolite ratios of RR and RC at different developmental stages in each sampling time was constructed. The results showed that the differences of metabolites between RR and RC were different in cultivation and at different stages. The effects of RR and RC on primary (Figure 4, Supplementary Table S3) and secondary metabolites (Figure 5, Supplementary Table S3) were further analyzed and compared. Compared with the RC group, there are 12 metabolites that decreased in the RR group at the different developmental stages, including four amino acids and derivatives, three organic acids, one other compound and four flavonoids. Among them, 2-aminoadipic acid, threonate, galloyl-Hexahydroxy dibenzoylglucose #1, galloyl-Hexahydroxy dibenzoylglucose #2, Hexahydroxy dibenzoyl digalloylglucose #1, and Hexahydroxy dibenzoyl digalloylglucose #2 were decreased in the grape pulp at all stages of RR. Forty metabolites increased in the RR group at the different developmental stages, including 22 amino acids and derivatives, six nucleotides, four carbohydrates, four cofactors, three cinnamic acids and one other compound. Among them, caftaric acid glutathione, glutamylleucine, proline, urate, *trans*-urocanate, 2′-deoxycytidine, phosphocholine, riboflavin, cyanidin-3-*O*-glucoside #3, quercetin-3-*O*-rhamnoside #1, epicatechin, chrysoeriol-*O*-hexoside, caffeic acid-*O*-hexoside #1, *trans*-2-Hydroxycinnamate, gallic acid, linoleoyl ethanolamide #1, and linoleoyl ethanolamide #2 in the RR group at all stages were higher than those in the RC group.

## 3. Discussion

The grape berry is a non-climacteric fruit that exhibits a double sigmoid growth pattern and undergoes a series of complex biochemical and physiological changes during the development and ripening stages [22,23]. The whole growth process can be divided into three distinct phases [24]. In the first stage, the berry size increases rapidly due to cell division and expansion, which is called the green stage; the berries are hard and green and accumulate organic acids (mainly malic and tartaric acids). This is followed by a lag stage, also known as hard-core stage, with little or no growth, but the grape berries begin to accumulate sugar. The last stage coincides with the onset of ripening; starting with the veraison, the berries begin to soften and the size continues to increase. Accompanying the berries’ growth, sugars (mainly glucose and fructose) accumulate rapidly, while the concentrations of organic acids and soluble tannins decrease. Furthermore, the grapes accumulate color (anthocyanins for red grapes) and gradually deepen. At the end of this stage, a large number of flavor compounds and volatile aromas are synthesized [23,25,26]. In addition, the composition and concentration of metabolites were prone to be influenced by biotic and abiotic stresses [9,27,28]. The variations in metabolic compositions affected by natural environments and external stresses relate to fruit quality and yield [29]. Therefore, it is significant to understand the metabolite changes at the metabolomic level during grape berry growth regarding the quality and yield improvement. In our study, we explored the kinetic patterns of the metabolite changes of grape pulp in response to RR from the hard-core stage to maturity by the non-targeted metabolomic method using UPLC-MS. Our metabolomic results showed that the metabolite changes of grape pulp are development-stage dependent, and revealed systematic influences of RR at the metabolomic level that could be used to find the reason for appearance and flavor differences between the RR and RC samples (Figure 1).

### 3.1. Primary Metabolites

Generally, grape berries’ quality depends mainly on the ratio of sugars to organic acids. Sugar, especially fructose and glucose, determine the sweetness and flavor of grape berries, which are hydrolyzed from the sucrose at a suitable pH [30]. The sucrose is the form of transport from the leaves through the phloem, whose content could be influenced by a disproportionate ratio of importing for consumption during the growth processes of grape berry [30,31,32]. Sugar accumulation in grape berries mainly depends on the activity of sugar-metabolizing enzymes, such as acid invertases, neutral invertase sucrose synthase and sucrose phosphate synthase [17,33], and their concentrations are easily affected by cultural management and environmental stresses [34]. Previous studies have shown that RR increased the number of plasmodesmata between sieve elements and companion cells and between the sieves’ elements/companion cells’ complex and phloem parenchyma cells [35] and distributed more dry material into berries [35]. Meanwhile, Xie et al. found that the change of the acid invertase activity has a similar trend to the change of total sugar content, which is presumed to be the critical enzyme for the RR treatment to promote the sucrose, glucose and fructose accumulation [17]. Duan et al. found that the nitrogen uptake and amino acid synthesis were inhibited in xylem sap under RR at the veraison and harvest stages, which might lead to the acceleration of more photosynthetic products distributed to berries and promote the sugar accumulation [36]. In our study, we also observed that glucose and fructose are the main sugars in our samples, and an obvious increase in their contents in the pulp of berries under the RR group compared with those in RC was noted, which was in agreement with the previous results (Figure 4). In addition to increasing grape berry sugars, some studies also found that RR can also reduce organic acids and increase amino acid contents [1,19]. The reduced organic acids in RR samples would promote amino acid biosynthesis to respond to RR stress [21].

In the present study, we found that amino acids biosynthesis and metabolism were differentially affected by the RR treatment in grape berry pulp (Figure 4). Proline as an osmotic adjustment substance is one of the most abundant amino acids in grape berry, which was significantly increased under biotic and abiotic stresses [37,38,39]. Our results showed that proline concentrations increased from the hard-core stage to maturity in both RR and RC samples, and there were significantly higher proline contents for the samples from the RR groups compared with those in the control groups (Figure 2 and Figure 4). Similar results were found in mango trees [40]. Therefore, the increase of proline content in RR samples can be used as an alternative biomarker for plant adaptation to RR stress [40]. Glutamate is a common intermediate in the biosynthesis of proline and glutamine, which increased in the grape berry pulp from the hard-core stage to maturity, and leads to an increase the proline content [41]. In our study, glutamine content increased at the earlier stages of grape growth but reduced at the later stages of grape growth under the RR, because of the accumulation of proline at the same time. In addition, aromatic amino acids (tryptophan, phenylalanine, and tyrosine) increased in the RR samples, because these aromatic amino acids could contribute to improve secondary metabolites and fruit quality (Figure 2 and Figure 4). On the other hand, several stress-responsive amino acids (2-aminoadipic acid, aspartate and serine) declined under RR treatment (Figure 4), because these amino acids are used for sugars and energy metabolism, or for conversion to other essential amino acids [42]. Among other primary metabolites, lipids metabolism can be considered as an indicator of environmental stresses [43], because it is involved in many biological processes, such as being structural components for cell membranes as phospholipid bilayers, providing structural and functional molecules for energy metabolism, and participating in signal transport [44]. Higher lipids content, especially lysoPEs, indicated that RR uses a specific lipid metabolism strategy as a potential biological mechanism for grape to fight against environmental variation. Meanwhile, the nucleotides contents accumulated in RR were higher than that in RC (Figure 4), indicating that the general responses of plants to other stresses were similar [21].

### 3.2. Secondary Metabolites

The grape berry growth processes involve a series of biochemical and biophysical changes [45]. As with most plants, the quality of grape berry mainly depends on its metabolites. The production of these metabolites is particularly sensitive to external conditions. Compared to primary metabolites, the chemical diversity of grape berry is mainly affected by secondary metabolites [46]. In general, secondary metabolites were reported to play critical physiological roles in plants, including adaptation to the environment, acquired resistance to pests, pollination, and symbiosis with microorganisms [47,48], and were also crucial in the determination of the quality of food attributes (taste, color and aroma) [4,49,50].

Previous studies have shown that RR could promote resveratrol, anthocyanins, carotenoids, phenols and flavonoids biosynthesis [18,20,21]. However, most of these studies have focused on the grape berry skins, and the main studies in the pulp tissues are primary metabolites, but the pulp also contains many secondary metabolites. Flavanols are an abundant class of flavonoids in grape berries with strong antioxidant activity, and responsible for the bitterness, aroma and astringent properties of foods and beverages [51]. In our study, RR significantly increased the levels of most flavanols, except for Proanthocyanidin B1 and Proanthocyanidin B2 in the pulp tissues at the maturity stage, which improves antioxidants to protect grape berries (Figure 5). However, from another perspective, it also adds to the astringency of the berry pulp. It is worth noting that RR reduces the concentrations of hydrolysable tannins, improving the flavor and taste of the berry pulp. Notably, most other classes, and some amines and fatty acyls, were increased at maturity in the pulp in RR but not RC (Figure 5). This could be the reason why grape berry in RR showed more flavor and aroma than those in RC. However, the changes of secondary metabolites in pulp appeared less significant compared with skin [21]. All the same, RR is a potential alternative cultivation for manufacturing practices.

## 4. Materials and Methods

### 4.1. Plant Material and Sample Collection

Three-year-old grapevines “Red Alexandria” under RR and RC cultivations during the fruiting season of 2016 to 2017 were used as the materials in the present study in a greenhouse of Shanghai Jiao Tong University, Shanghai, China. Grapevines with the RR treatment were planted in plastic boxes (60 cm × 45 cm × 45 cm), while the RC treatment were planted in a 45-cm-deep raised bed with the same medium (a mixture of sand, loam and manure, 1:1:1, *v/v/v*) in open ground. The same watering and fertilizer strategies were applied to the RR and RC groups to avoid different environmental conditions.

The grape berries were collected at five different developmental stages, namely S1 (hard-core stage, eight weeks after full bloom, WAFB), S2 (pre-veraison stage, 10 WAFB), S3 (veraison stage, 12 WAFB), S4 (pre-ripening stage, 14 WAFB) and S5 (harvest-ripe stage, 16 WAFB), respectively. About 10 clusters were randomly picked from at least five individual plants at each sampling. Three biological replicates were set for both RR and RC, and 10 berries were randomly selected from a pool of berries for each biological replicate. All samples were picked and selected for uniform maturity and the absence of disease or mechanical damage. All grape pulp were separated from skin, cut into small pieces and then frozen in liquid nitrogen immediately, ground into powder and stored at −80 °C for future use.

### 4.2. Metabolite Extraction and Profiling

About 50 mg of grape pulp was extracted with 40 volumes (*w/v*) of ice-cold methanol by sonication at 40 Hz for 30 min at room temperature [21,52]. The extracts were centrifuged at 12,000 rpm for 10 min at 4 °C. The supernatant was collected, and the precipitate was re-extracted twice, as above. All the supernatants were combined and filtered through 0.2-μm-diameter-pores membrane (Nylon) before injecting into the UPLC-MS system.

The UPLC analysis was performed using an Agilent 1290 Infinity II LCTM system, coupled with an Agilent Eclipse Plus C18 column (3.0 mm × 150 mm, 1.8 μm). The compounds were eluted with mobile phase A (0.1% formic acid-water) and mobile phase B (100% acetonitrile) at a flow rate of 0.4 mL/min. The gradient elution was as follows: 0 to 1 min, 98% A; 1 to 5 min, 98 to 60% A; 5 to 12 min, 60 to 30% A; 12 to 15 min, 30 to 5% A; 15 to 20 min, 5% A. The column temperature was set at 40 °C and the injection volume was 10 μL. Mass spectrometry (MS) analysis was carried out by using an Agilent 6550 iFunnel/Q-TOF System equipped with an Agilent Jet-Stream source. The optimal MS conditions were as follows: scan range *m/z* 50 to 1000; both positive and negative ionization mode; 16 L/min for drying gas and 25 psi for nebulizer heated at 350 °C. The capillary voltage of the positive mode and negative mode were 3500 V, nozzle voltage at 1500 V (−), and 250 V (+); fragmentor voltages at 380, 10, 20 and 40 V were applied to collision-induced dissociation voltage. Detailed information on data collection is referred to in a previous study [53]. Metabolite annotations were performed by searching the Personal Compound Database and Library (PCDL), Metlin database (available online: http://metlin.scripps.edu (accessed on 8 October 2017)) [54], Massbank database (available online: http://www.massbank.jp/en/manual.html (accessed on 15 October 2017)) [55] and the data reported in the literature [30]. MassHunter Acquisition 6.0, MassHunter Qualitative 6.0, and Mass Profinder 6.0 were used for data acquisition, review, and peak area extraction, respectively.

### 4.3. Statistical Analysis

The data was normalized as reported previously [53]. The peak area was used to quantify the abundance of metabolites, which was divided by the median value of each metabolite and the weight of the sample. The results are the mean ± SE of at least three independent replicates and the principal component analysis (PCA) were analyzed using SIMCA-P version 11.0. The statistical significance of differences was determined with a *t*-test, and the false discovery rate (FDR) was used for multiple testing correlation (FDR < 0.05). The heatmaps were generated using MultiExperiment Viewer version 4.8.

## 5. Conclusions

In the present study, our results show that 283 metabolites were annotated in developing berry pulp tissues using a non-targeted metabolomics approach and compared the metabolomics kinetics of developing berry pulp tissues under RR and RC conditions. Principal component analysis showed that the effect of the development period on pulp metabolites was greater than that of cultivation methods. We found that 12 metabolites were decreased and 40 metabolites were increased in the RR group at the different developmental stages compared with the RC group. In summary, we found that RR can improve grape fruit quality by through primary and secondary metabolite analysis at different developmental stages.

## Data Availability

The data presented in this study are available in article and Supplementary Materials.

## Supplementary Materials

The following are available online at https://www.mdpi.com/article/10.3390/metabo11060377/s1, Table S1: List of metabolites annotated in developing grape pulp, Table S2: The ratios of metabolite changes in pulp tissues of developing berries for each cultivation method as compared with S1 stage, Table S3: The ratios of metabolite changes in pulp tissues of developing berries under RR condition as compared with those in RC.
